# Metabolic syndrome is associated with change in subclinical arterial stiffness - A community-based Taichung Community Health Study

**DOI:** 10.1186/1471-2458-11-808

**Published:** 2011-10-17

**Authors:** Chia-Ing Li, Sharon LR Kardia, Chiu-Shong Liu, Wen-Yuan Lin, Chih-Hsueh Lin, Yi-Dar Lee, Fung-Chang Sung, Tsai-Chung Li, Cheng-Chieh Lin

**Affiliations:** 1 Department of Medical Research, China Medical University Hospital, Taichung, Taiwan; 2 School of Medicine, College of Medicine, China Medical University, Taichung, Taiwan; 3 Department of Public health, College of Public Health, China Medical University, Taichung, Taiwan; 4 Department of Epidemiology, University of Michigan, Ann Arbor, Michigan, USA; 5 Department of Family Medicine, China Medical University Hospital, Taichung, Taiwan; 6 Department of Psychiatric, Medical College, National Cheng-Kung University, Tainan, Taiwan; 7 Bristol-Myers Squibb Ltd, Global Development & Medical Affair, Taipei, Taiwan; 8 Institute of Biostatistics, China Medical University, Taichung, Taiwan; 9 Department of Healthcare Administration, College of Health Science, Asia University, Taichung, Taiwan

**Keywords:** metabolic syndrome, pulse wave velocity, arterial stiffness

## Abstract

**Background:**

The aim of this study was to evaluate the effect of MetS on arterial stiffness in a longitudinal study.

**Methods:**

Brachial-ankle pulse wave velocity (baPWV), a measurement interpreted as arterial stiffness, was measured in 1518 community-dwelling persons at baseline and re-examined within a mean follow-up period of 3 years. Multivariate linear regression with generalized estimating equations (GEE) were used to examine the longitudinal relationship between MetS and its individual components and baPWV, while multivariate logistic regression with GEE was used to examine the longitudinal relationship between MetS and its individual components and the high risk group with arterial stiffness.

**Results:**

Subjects with MetS showed significantly greater baPWV at the end point than those without MetS, after adjusting for age, gender, education, hypertension medication and mean arterial pressure (MAP). MetS was associated with the top quartile of baPWV (the high-risk group of arterial stiffness, adjusted odds ratio [95% confidence interval] 1.52 [1.21-1.90]), and a significant linear trend of risk for the number of components of MetS was found (p for trend < 0.05). In further considering the individual MetS component, elevated blood pressure and fasting glucose significantly predicted a high risk of arterial stiffness (adjusted OR [95% CI] 3.72 [2.81-4.93] and 1.35 [1.08-1.68], respectively).

**Conclusions:**

MetS affects the subject's progression to arterial stiffness. Arterial stiffness increased as the number of MetS components increased. Management of MetS is important for preventing the progression to advanced arterial stiffness.

## Background

Metabolic syndrome (MetS), defined as a cluster of features such as visceral obesity, impaired glucose tolerance, dyslipidemia, hypergtriglyceridemia, and elevated blood pressure [1,2], is highly prevalent all over the world [3-12]. MetS has been known as a critical risk factor in the incidence of type 2 diabetes and in cardiovascular outcome [1,13,14]. People with MetS have higher all-cause or cardiovascular mortality than those without MetS [15-17]. Arterial stiffness, a pathological condition with vascular damage, is a cardiovascular outcome of MetS. In clinical practice, pulse wave velocity (PWV) is widely used to reflect arterial stiffness. A noninvasive brachial-ankle pulse wave velocity (baPWV) measurement, which is performed more easily than carotid-femoral PWV measurement, has been used as a marker for screening vascular damage and cardiovascular risk in the general population [18,19], in diabetes patients [20,21], in hypertension patients [22,23], in patients with end-stage renal disease [24,25], and in women with systemic lupus erythematosus [26].

The association of MetS with arterial stiffness has been investigated in many studies [27-31]; however, most of these studies were cross-sectional [27-29,31] Studies explored the longitudinal effect of MetS on arterial stiffeness in specific subjects, such as patients who were systemic lupus erythematosus [32], patients who were newly detected suspected hypothyroidism [33], and persons who received work-related health check-up [34] or general health check-up [35]. None of them was community-based study. One study reported a longitudinal relation between PWV and MetS only in men workers [34]. Another 6-year follow-up study perfomed by Safar et al explored this relationship in subjects selected from receiving general health check-up population with prevalence of elevated blood pressure of 50% [35]. Moreover, high prevalence of diabetes and dyslipidemia in the study by Safar et al was found. It is very likely to have Berkson's bias in their study [36]. Previous study showed the association may be found significantly in clinical-based studies, but not in community-based studies [37]. Our study is the first community-based study using the probability sampling method to select a random sample from a well-defined population. Identifying the effect of MetS on arterial stiffness using a longitudinal study in a community-based population can provide information for the management of MetS and thereby prevent progression to advanced arterial vascular disease. Therefore, the objective of the current study was to evaluate the longitudinal effect of MetS on baPWV by considering mean arterial pressure (MAP) and the use of hypertension medicine to reduce the influence of blood pressure on baPWV.

## Methods

### Study sample

This was a population-based follow-up study. The design and selection criteria of the Taichung Community Health Study (TCHS) have been described previously [4]. Briefly, 4, 280 individuals were randomly selected from Taichung City, Taiwan to be representative of its residents in terms of sex and age (aged 40 and over). Data on 2, 359 individuals (about 66.83% of the original sample) were collected from 2004-2005, and 2, 311 survivors were contacted 3 years later for re-examination. A total of 1, 648 subjects were followed (overall follow-up rate, 71.3%) before the end of July, 2009. Of those, 28 were excluded from this analysis because they did not have a baPWV measurement at baseline or follow-up, 100 were excluded because they had suspected peripheral arterial stiffness (ankle-brachial index < 0.9 at baseline), and 2 were excluded because of missing smoking status at baseline. In the end, 1, 518 individuals (mean age, 56 years; 49% women) remained eligible for data analysis. All subjects signed an informed consent form before data collection.

## Measurements

### Anthropometric measurements and laboratory examination

All study subjects underwent a physical examination measuring height, waist circumference (WC), and blood pressure by trained staff at baseline, as well as at the endpoint. MAP was calculated as (2 ×diastolic blood pressure + systolic blood pressure) ÷ 3. Blood was drawn with minimal trauma from an antecubital vein in the morning, after a 12-hour overnight fasting, and was sent for analysis within four hours of collection. Biochemical markers such as HDL cholesterol, triglyceride, and fasting glucose were analyzed by a biochemical autoanalyzer (Beckman Coluter, Lx-20, USA) at the Clinical Laboratory Department of China Medical University Hospital.

### Sociodemographic factors, lifestyle factors and medical history

Data on sociodemographic factors (including age, gender and education), lifestyle factors, and medical history were collected by self-administered questionnaires. Lifestyle factors, such as smoking and alcohol drinking history, were categorized as never and former/current. We collected self-reported personal medical histories, including diabetes and hypertension medication at the baseline and follow-up examination.

### MetS and its components

MetS was defined clinically, based on the presence of three or more of the following American Heart Association and the National Heart Lung Blood Institute(AHA/NHLBI) MetS criteria [2]: (1) central obesity (WC ≥ 90 cm in men, and ^3^80 cm in women), (2) high triglycerides level (^3^1.7 mmol/L or on drug treatment for elevated triglycerides), (3) low HDL-C level (< 1.03 mmol/L in men and < 1.30 mmol/L in women or on drug treatment for reduced HDL-C), (4) high blood pressure (systolic BP ^3^130 mmHg or diastolic BP ^3^85 mmHg or under anti-hypertensive drug treatment in a patient with a history of hypertension), and (5) high fasting plasma glucose concentration (^3^5.5 mmol/L or on drug treatment for elevated glucose). Diabetes was defined as fasting plasma glucose concentration ^3^7.0 mmol/L or on drug treatment for diabetes. Hypertension was defined as systolic BP ^3^140 mmHg or diastolic BP ^3^90 mmHg or on drug treatment for hypertension.

### Longitudinal changes in arterial stiffness

BaPWV, presenting for arterial stiffness, was measured non-invasively with subjects in the supine position and with a VP-1000 automated PWV/ABI analyzer (PWV/ABI; Colin Co. Ltd., Komaki, Japan) attached to the four limbs [38]. For every subject, the maximum of the left and right baPWV was chosen at baseline and at follow-up. The change in baPWV was calculated as re-examined baPWV subtracting baseline baPWV.

### Statistical analysis

Continuous variables were reported as mean ± standard deviation (SD) and categorical variables were reported as percentage (95% confidence intervals, abbreviated as CI). The variables that predicted the baPWV change were evaluated by analysis of covariate with baPWV at baseline as covariate due to its high impact on the baPWV change. Moreover, to explore the effect of MetS and its components on baPWV, three multivariate models were used. First, the longitudinal effect of MetS and the number of components on the change baPWV at follow-up were examined using multivariate linear regression with the generalized estimating equations (GEE) method. Second, we further evaluated how the longitudinal effect of individual MetS components on baPWV was affected by the other components being considered sequentially, using hierarchical linear regression analysis with the GEE approach. The order of entering the variables was elevated blood pressure, fasting glucose, WC, triglyceride, and low HDL cholesterol after adjustment. Last, the top quartile of baseline baPWV was used as the cutoff point to classify the high risk group with arterial stiffness. Multivariate logistic regression with the GEE approach was used to analyze the longitudinal effect of MetS and its components on arterial stiffness. We treated the number of MetS components as continuous variables to examine the linear trend on the risk of arterial stiffness. All reported *p *values were those of the two-sided tests; statistical significance was set at *p *< 0.05. All analyses were performed using SAS version 9.1 (SAS Institute Inc, Cary, NC).

## Results

Compared with individuals without MetS, a higher proportion of individuals with MetS were older, male, with ≤ 9 years of educational attainment, former or current smokers, former or current drinkers, and users of hypertension, hyperlipidemia, and diabetes medication (Table 1).

**Table 1:** Baseline characteristics of subjects with and without metabolic syndrome

	Non-MetS^†^		MetS^†^		
	
Variable at baseline	n	(%)	n	(%)	p value
**Sociodemographic factors**					
Age					< 0.001
40-50 years	372	(39.2)	128	(22.5)	
51-60 years	335	(35.3)	187	(32.9)	
61-70 years	151	(15.9)	140	(24.7)	
> 70 years	92	(9.6)	113	(19.9)	
Gender					< 0.001
Female	521	(54.8)	227	(40.0)	
Male	429	(45.2)	341	(60.0)	
Education					< 0.001
≤ 9 years	268	(28.3)	231	(40.7)	
10-12 years	471	(49.7)	256	(45.2)	
> 12 years	208	(22.0)	80	(14.1)	
Married status					0.691
Not currently married	154	(16.3)	87	(15.4)	
Married	790	(83.7)	478	(84.6)	
**Lifestyle factors**					
Smoking					< 0.001
Never	744	(78.3)	377	(66.4)	
Former/Current	206	(21.7)	191	(33.6)	
Drinking					0.008
Never	691	(72.7)	376	(66.2)	
Former/Current	259	(27.3)	192	(33.8)	
**Medication**					
Hypertension					
Yes	139	(14.6)	265	(46.7)	< 0.001
Hyperlipidemia					
Yes	51	(5.4)	132	(23.2)	< 0.001
Diabetes					
Yes	20	(2.1)	103	(18.1)	< 0.001

† MetS, metabolic syndrome

The baseline baPWV and changes in baPWV during the follow-up period, according to the various groups of sociodemographic factors, health factors, and medication after adjusting for baPWV at baseline, are illustrated in Table 2. Larger increases in changes of baPWV were observed in those with an older age, lower educational level, and use of medication for hypertension, hyperlipidemia, and diabetes at baseline (all *p *< 0.05). The changes were not significantly different in terms of marital status and health factors at baseline, such as smoking and drinking.

**Table 2:** Brachial-ankle pulse wave velocity (baPWV) at baseline and its changes adjusted for baPWV at baseline during the period of follow-up of baseline characteristics

		baPWV at baseline		Changes of baPWV during follow-up period*		
		
Variable at baseline	n	Mean	± SD	Adjusted mean ± SE		p value
**Sociodemographic factors**						
Age						**< 0.001**
40-50 years	500	1365.1	± 212.7	-62.6	± 10.8	
51-60 years	522	1552.2	± 281.5	-18.4	± 9.6	
61-70 years	291	1789.3	± 366.2	70.9	± 13.2	
> 70 years	205	2147.2	± 459.2	141.0	± 18.1	
Gender						0.101
Female	770	1674.4	± 397.6	-3.8	± 8.3	
Male	748	1556.6	± 399.3	14.9	± 8.2	
Education						**0.004**
≤ 9 years	499	1736.7	± 443.3	34.3	± 10.2	
10-12 years	727	1566.5	± 372.9	-6.7	± 8.4	
> 12 years	288	1532.1	± 353.0	-10.3	± 13.3	
Married status						0.398
Not currently married	241	1660.5	± 446.4	17.0	± 14.6	
Married	1268	1607.3	± 393.3	3.6	± 6.3	
**lifestyle factors**						
Smoking						0.346
Never	1121	1603.1	± 415.1	2.4	± 6.7	
Former/Current	397	1653.9	± 363.2	14.9	± 11.3	
Drinking						0.553
Never	1067	1623.6	± 416.8	7.9	± 6.9	
Former/Current	451	1599.4	± 367.0	0.4	± 10.6	
**Medication**						
Hypertension						**< 0.001**
No	1197	1541.9	± 355.3	-8.6	± 6.6	
Yes	304	1903.1	± 440.7	68.8	± 13.6	
Hyperlipidemia						**0.002**
No	1401	1604.9	± 397.8	1.3	± 6.0	
Yes	93	1779.7	± 397.3	77.7	± 23.3	
Diabetes						**< 0.001**
No	1415	1591.3	± 382.0	-3.8	± 5.9	
Yes	94	1997.6	± 476.7	146.7	± 23.7	

SD, standard deviation; SE, standard error

* The difference in each variables was calculated by analysis of covariance with baPWV at baseline as covariate.

Subjects with MetS had a higher mean value of baPWV at baseline. Compared to subjects without MetS, subjects with MetS had a significantly greater 3-year mean change of baPWV (-9.5 vs. 31.1, after adjusting for baPWV at baseline) (Table 3). The greater the number of MetS components at baseline, the larger the adjusted mean changes of baPWV (*p *for trend < 0.001).

**Table 3:** Brachial-ankle pulse wave velocity (baPWV) at baseline and its changes with baPWV at baseline as covariate according to MetS status and the number of MetS components

		baPWV at baseline		3-year changes of baPWV^†^			
		
Variable at baseline	n	Mean	± SD	Adjusted mean ± SE		*p *value	Trend test *p *value
MetS						0.001	-
No	950	1518.5	± 373.6	-9.5	± 7.4		
Yes	568	1780.1	± 396.6	31.1	± 9.7		
Number of MetS components						< 0.001	< 0.001
0	221	1342.2	± 203.0	-37.2	± 15.7		
1	354	1499.6	± 380.6	-16.2	± 12.1		
2	375	1640.3	± 399.5	10.8	± 11.6		
≥ 3	568	1780.1	± 396.6	32.6	± 9.8		

MetS, metabolic syndrome; SD, standard deviation; SE, standard error.

^†^All values were calculated by analysis of covariance with baPWV at baseline as covariate.

**Figure 1:**
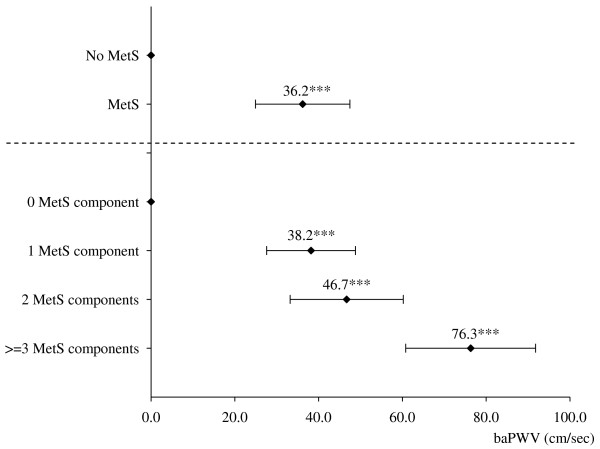
**Multivariate linear regression with the use of generalized estimating equations of longitudinal brachial-ankle pulse wave velocity (baPWV) based on metabolic syndrome (MetS) and its number of components**. Both models were adjusted for age, gender, education, smoking status at baseline, time-dependent hypertension medication, and time-dependent mean arterial pressure. Point estimates and standard errors of baPWV for MetS and each number of MetS components were shown as rhombic point and horizontal bar. ***p < 0.001

Analysis of the longitudinal effect of MetS on baPWV using GEE models showed that subjects with MetS had a higher mean baPWV of 36.2 (*p *< 0.001) after adjusting for age, gender, education, smoke status, time-dependent hypertension medication, and time-dependent MAP (Figure 1). Considering the longitudinal effect of the number of MetS components on baPWV, the differences in baPWV in subjects with 1, 2 and more than or equal to 3 MetS components versus those without a MetS component were 38.2, 46.7 and 76.3, respectively (all *p *< 0.001) (Figure 1).

In exploring the independent effects of MetS components, hierarchical regression analysis with the GEE approach demonstrated that elevated blood pressure (adjusted regression coefficients [β] = 28.3, *p *< 0.05) and fasting glucose (b = 51.4, *p *< 0.001) had an independent effect on longitudinal baPWV (Table 4).

**Table 4:** Hierarchical multivariate linear regression with generalized estimating equation analysis for brachial-ankle pulse wave velocity (baPWV) changes in 1518 subjects during a 3-year follow up

	Estimate (95% CI)				
	
MetS† components	Model 1	Model 2	Model 3	Model 4	Model 5
Elevated blood pressure	32.3* (6.2~58.3)	29.2* (3.4~55.0)	29.8* (4.1~55.6)	28.2* (2.5~53.9)	28.3* (2.5~54.0)
Elevated fasting glucose		51.6*** (31.7~71.4)	52.5*** (32.5~72.6)	51.5*** (31.5~71.5)	51.4*** (31.3~71.4)
Central obesity			-9.5 (-30.9~11.9)	-11.1 (-32.6~10.4)	-11.3 (-32.8~10.2)
Elevated triglyceride				20.8 (-1.8~43.4)	20.2 (-2.6~43.0)
Low HDL^† ^cholesterol					2.7 (-16.9~22.3)

CI, confidence interval.

^† ^MetS, metabolic syndrome; HDL, high-density lipoprotein

All models were adjusted for age, gender, education, smoking status at baseline, time-dependent hypertension medication, and time-dependent mean arterial pressure.

* p < 0.05, ** p < 0.01, *** p < 0.001

**Figure 2:**
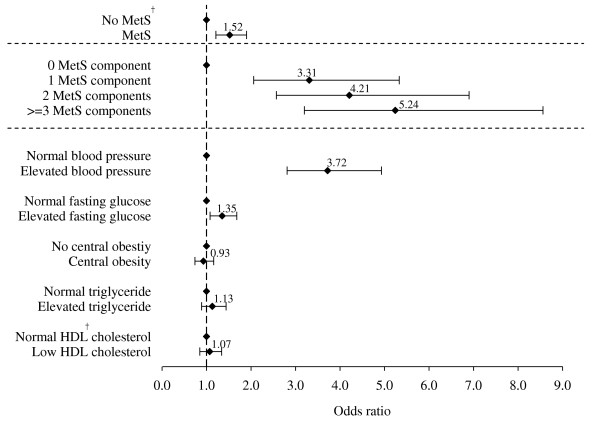
**Multivariate-adjusted odds ratio of top quartile of brachial-ankle pulse wave velocity (baPWV) at baseline based on MetS, number of MetS components, and individual MetS components after adjusting for age, gender, education, smoking status at baseline, time-dependent hypertension medication, and time-dependent mean arterial pressure**. The cut-off point of the top quartile of baseline baPWV was 1813 cm/s. ^† ^MetS, metabolic syndrome; HDL, high-density lipoprotein. * The linear trend of baPWV changes in the number of MetS components in the top quartile was statistically significant.

The cutoff point of the top quartile of baseline baPWV was 1813 cm/s. The adjusted odds of the high-risk group with arterial stiffness for MetS were 1.52 (95% confidence interval [CI], 1.21-1.90). The adjusted odds ratios (ORs) of the high-risk group with arterial stiffness were 3.31 (95% CI, 2.06-5.33), 4.21 (95% CI, 2.57-6.91), and 5.24 (95% CI, 3.20-8.57) in subjects with 1, 2, and ≥ 3 MetS components after multivariate adjustment. A significant linear relationship between the adjusted OR and the number of components of MetS was found (*p *for trend < 0.05). The adjusted ORs of the high-risk group with arterial stiffness were 3.72 (95% CI, 2.81-4.93) and 1.35 (95% CI, 1.08-1.68) in individuals with elevated blood pressure and fasting glucose (both *p *< 0.05) (Figure 2).

## Discussion

In this 3-year community-based prospective study, we found that MetS and the MetS components independently predicted the future progression or incidence of arterial stiffness. Regarding the influence of individual components of MetS on arterial stiffness, we found that blood pressure and fasting glucose were independent determinants of longitudinal arterial stiffness progression in this general population. Moreover, they were independent risk factors for predicting the 3-year incidence of the higher risk of developing arterial stiffness.

The existence of a strong association between the presence of MetS and arterial stiffness has been shown in many cross-sectional studies [27-29]. However, the longitudinal effect of MetS on arterial stiffness in the general population has not been clarified [34,35,39]. In this longitudinal population-based study, we provided community-based evidence that MetS is an independent risk factor for progression of arterial stiffness in a random sample of Taiwaneses adults aged 40 years and over. Our findings were consistent with those of several previous studies that showed a causal relationship between MetS and arterial stiffness. In a 3-year prospective study of 2, 080 male employees in a company, Tomiyama et al. showed that subjects with persistent MetS had a higher annual rate of increase in baPWV than those with regression of MetS during the follow-up period [34]. Similar deleterious effects of MetS on aortic stiffness were found in a 6-year follow-up study of a health check-up for 476 French adults who were working and retired persons and their families [35].

In the present study, only elevated blood pressure and fasting glucose were independent predictors of progressive arterial stiffness. Tomiyama et al. [34], in their study of 2080 male employees aged 29 to 76 years with an average of 3 years of follow-up, found elevated blood pressure and fasting glucose at baseline were independent predictors for changes in baPWV, which was consistent with our findings. Li et al [39] followed 835 young adults aged 4 to 17 years with 26.5 years in average, and found that the independent predictors of baPWV in young adults were systolic blood pressure, HDL cholesterol, and triglycerides in adulthood. However, we did not find that elevated triglyceride and low HDL cholesterol were associated with longitudinal baPWV. There are two possible reasons why Li et al.'s findings [39] were not consistent with ours. One is that they did not measure baPWV at baseline and could not correct or adjust it, which may have resulted in overestimating the effects of triglyceride and HDL cholesterol. The other is that our study lacked power to detect the effect of triglyceride and HDL cholesterol, due to the shorter follow-up period than that in Li et al's study [39].

The possible mechanism that can explain the effect of elevated blood pressure on progressive arterial stiffness is its direct effect on arterial walls. Elevated blood pressure may accelerate arterial stiffening because it forces endothelial cells and arterial smooth muscle cells to be exposed to the increase arterial wall dispensability chronically, which reflects arterial stiffening [40]. Elevated blood glucose leads to the formation and deposition of advanced glycation end-products, which promote the crosslinking of collagen that stiffens the structural components of the arterial wall [41].

This study has several strengths. First, this was a prospective study; therefore, the temporal relationship between metabolic risk factors and arterial stiffness could be clearly ascertained. Second, this was a community-based cohort which could be representative of the general population. And lastly, our study evaluated arterial stiffness using non-invasive and simple baPWV measurement, and statistical corrections were made to prevent the influence of blood pressure. However, there are two limitations to our study. One is that our findings could not be generalized to young adults because we recruited participants aged 40 and over. The other is that the findings of our study may not be generalized to adults living in areas of less urbanization, because our sample was randomly selected from a population in a metropolitan area.

## Conclusions

We found that MetS and its components of fasting glucose and blood pressure are independent predictors of the longitudinal increase in arterial stiffness. Since these predictors are associated with significant cardiovascular morbidity and mortality, our findings suggest that management of MetS to prevent progression to advanced arterial vascular disease is important.

## Competing interests

The authors declare that they have no competing interests.

## Authors' contributions

CCL and TCL conceived and designed the experiments. CIL, TCL and CSL analyzed the data. WYL, CHL, YDL, and CCL participated in coordination and evaluation of data. SLK and FCS contributed to the study with their knowledge on field study. CCL, TCL and CCL wrote the paper. All authors read and approved the final manuscript.

## Pre-publication history

The pre-publication history for this paper can be accessed here:

http://www.biomedcentral.com/1471-2458/11/808/prepub
